# Infection of XC Cells by MLVs and Ebola Virus Is Endosome-Dependent but Acidification-Independent

**DOI:** 10.1371/journal.pone.0026180

**Published:** 2011-10-12

**Authors:** Haruka Kamiyama, Katsura Kakoki, Hiroaki Yoshii, Masatomo Iwao, Tsukasa Igawa, Hideki Sakai, Hideki Hayashi, Toshifumi Matsuyama, Naoki Yamamoto, Yoshinao Kubo

**Affiliations:** 1 Department of AIDS Research, Institute of Tropical Medicine, Global COE Program, Nagasaki University, Nagasaki, Japan; 2 Graduate School of Science and Technology, Nagasaki University, Nagasaki, Japan; 3 Department of Urology, Graduate School of Biomedical Sciences, Nagasaki University, Nagasaki, Japan; 4 Division of Cytokine Signaling, Graduate School of Biomedical Sciences, Nagasaki University, Nagasaki, Japan; 5 Department of Microbiology, National University of Singapore, Singapore, Singapore; University of Minnesota, United States of America

## Abstract

Inhibitors of endosome acidification or cathepsin proteases attenuated infections mediated by envelope proteins of xenotropic murine leukemia virus-related virus (XMRV) and Ebola virus, as well as ecotropic, amphotropic, polytropic, and xenotropic murine leukemia viruses (MLVs), indicating that infections by these viruses occur through acidic endosomes and require cathepsin proteases in the susceptible cells such as TE671 cells. However, as previously shown, the endosome acidification inhibitors did not inhibit these viral infections in XC cells. It is generally accepted that the ecotropic MLV infection in XC cells occurs at the plasma membrane. Because cathepsin proteases are activated by low pH in acidic endosomes, the acidification inhibitors may inhibit the viral infections by suppressing cathepsin protease activation. The acidification inhibitors attenuated the activities of cathepsin proteases B and L in TE671 cells, but not in XC cells. Processing of cathepsin protease L was suppressed by the acidification inhibitor in NIH3T3 cells, but again not in XC cells. These results indicate that cathepsin proteases are activated without endosome acidification in XC cells. Treatment with an endocytosis inhibitor or knockdown of dynamin 2 expression by siRNAs suppressed MLV infections in all examined cells including XC cells. Furthermore, endosomal cathepsin proteases were required for these viral infections in XC cells as other susceptible cells. These results suggest that infections of XC cells by the MLVs and Ebola virus occur through endosomes and pH-independent cathepsin activation induces pH-independent infection in XC cells.

## Introduction

Murine leukemia viruses (MLVs) are divided into four groups according to their host ranges. Ecotropic MLV (Eco-MLV) infects mouse and rat cells. Amphotropic MLV (Ampho-MLV) infects many types of mammals including mouse, rat, mink, and human. The Eco- and Ampho-MLVs recognize cationic amino acid transporter 1 (CAT1) [1] and phosphate symporter 2 (Pit2) [2], [3], [4] as the infection receptors, respectively. Polytropic (Poly-) and xenotropic (Xeno-) MLVs both utilize the cell surface receptor protein XPR as the infection receptor [5], [6], [7]. The Poly- and Xeno-MLVs infect many types of mammals; however, the latter does not infect laboratory mice.

The MLV entry into the host cell cytoplasm occurs through membrane fusion between the viral envelope and host cell membranes. This membrane fusion is induced by viral envelope (Env) glycoproteins. The MLV Env protein has 16-amino acids at its C-terminal tail that is cleaved during virion maturation. The C-terminal proteolytic fragment is referred to as the R peptide. The R peptide-truncated Eco-MLV Env protein induces membrane fusion, while the full-length Env protein lacks this activity, indicating that the R peptide inhibits membrane fusion [8], [9], [10]. This membrane fusion activity enables proteolytically cleaved Env-expressing cells to fuse with neighboring susceptible cells, which may reflect the viral entry process.

Endosomal acidification also plays a role in the MLV infectious cycle. The Eco- and Ampho-MLV infections are suppressed by endosome acidification inhibitors that induce a rise in the pH of endosomes, showing that these viral infections require endosome acidification and occur through acidic endosomes [11], [12]. However, it has not been elucidated to date whether infections by the Poly- and Xeno-MLVs occur through acidic endosomes. A potential mechanism explaining the requirement for endosome acidification has recently been reported. Endosomal cathepsin proteases B and L are involved in the Eco-MLV infection [13], [14], and the cathepsin proteases are activated by low pH in acidic endosomes. Therefore, endosome acidification inhibitors may suppress the Eco-MLV infection by attenuating cathepsin protease activation. It is unknown whether cathepsins B and L play a role(s) in the Ampho-, Poly- and Xeno-MLV infections.

XC cells were established from a rat muscle tumor induced by Rous sarcoma virus [15], and are widely used to titrate Eco-MLVs, as the number of plaques resulting from the Eco-MLV-induced cell-cell fusion correlates with the viral titer [16]. It is thought that the mechanism of Eco-MLV entry into rat XC cells is distinct from other susceptible cells. pH-independent cell-cell fusion is induced in XC cells by the Eco-MLV infection [16], or by the R peptide-containing Eco-MLV Env protein [17], [18], but not in other susceptible cells. In addition, ammonium chloride, which increases the pH of endosomes, inhibits the Eco-MLV infection in many susceptible cells, but not in XC cells, indicating that the Eco-MLV infection in XC cells is independent of low pH in endosomes [11]. Therefore, it is generally accepted that the Eco-MLV enters XC cell at the plasma membrane, and enters other susceptible cells through acidic endosomes [11], [19].

In this study, we investigated whether the Poly-, Xeno-, and XMRV-MLV vector infections occur through acidic endosomes and whether these infections require cathepsin protease activity. We also examined the pH-independent mechanism of MLV infections in XC cells. We found that the Poly- Xeno-, and XMRV-MLV vector infections occur through acidic endosomes and require cathepsin proteases similar to the Eco-MLV, Ampho-MLV, and Ebola virus infections. The endosome acidification inhibitors attenuated all these viral infections in NIH3T3 and TE671 cells, but had no effect in XC cells, as previously shown [11]. The endosome acidification inhibitors attenuated cathepsin protease activities in TE671 cells, but had no effect in XC cells, indicating that cathepsin proteases are activated without endosome acidification in XC cells. Suppression of endocytosis by a dynamin GTPase inhibitor, dynasore, or by siRNA-mediated knockdown of dynamin 2 expression attenuated the MLV infections in all cells examined (including XC cells), showing that these infections occur through endosomes even in XC cells. These results show that cathepsin proteases are activated in the absence of endosome acidification in XC cells and that pH-independent virus infections occur through endosomes in these cells.

## Results

### Eco-MLV infection of XC cells is independent of endosomal acidification

**Figure 1:**
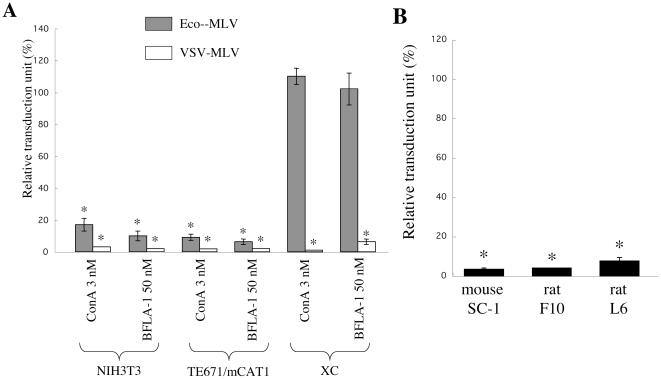
Eco-MLV infection in XC cells is independent of endosome acidification. (A) Relative titers of the Eco- or VSV-MLV vector on ConA- or BFLA-1-treated NIH3T3, TE671/mCAT1, and XC cells were indicated. (B) Relative titers of the Eco-MLV vector on ConA-treated mouse SC-1, rat F10, and L6 cells were indicated. The transduction units obtained with DMSO-treated cells were set to 100%. These experiments were repeated in triplicate, and results are shown as the mean +/− SD. Asterisks indicate statistically significant differences compared to the value from the DMSO-treated cells.

Ammonium chloride, which acts as a weak base and inhibits the acidification of endosomes, attenuates the Eco-MLV infection in NIH3T3 cells but not in XC cells [11]. This result suggests that the Eco-MLV entry into XC cells occurs at the plasma membrane and does not depend on endosome acidification. To test whether endosome acidification is required for the Eco-MLV infection in XC cells, we monitored the effects of concanamycin A (ConA) and bafilomycin A-1 (BFLA-1) on this process. MLV vectors carrying LacZ gene as a marker were pseudotyped with various viral envelope glycoproteins as described in Materials and Methods. Mouse NIH3T3, rat XC, and human TE671 cells expressing the Eco-MLV receptor (TE671/mCAT1) [14] were pretreated with the inhibitors for 5 h, and were inoculated with the Eco- or VSV-pseudotyped MLV vector diluted with fresh medium in absence of the inhibitor to minimize the effects on other steps of the MLV vector infection than entry (such as uncoating and reverse transcription). Treatment of NIH3T3 and TE671/mCAT1 cells with the inhibitors attenuated the Eco- and VSV-MLV vector infections (Fig. 1A). The treatment of XC cells inhibited the VSV-MLV vector infection, indicating that the treatment with ConA or BFLA-1 inhibited endosome acidification in XC cells. However, the treatment of XC cells did not attenuate the Eco-MLV vector infection as reported [11]. These results show that the Eco-MLV infection in XC cells does not require endosome acidification, while NIH3T3 and TE671/mCAT1 cells require endosome acidification for the infection.

XC cells are a rat cell line. We analyzed the effect of ConA treatment on the Eco-MLV infection in other rat cell lines (F10 and L6) as well as a mouse cell line (SC-1). The ConA treatment of all these cell lines efficiently inhibited the Eco-MLV vector infection (Fig. 1B), indicating that XC cells are unique cells in which the Eco-MLV infection is pH-independent.

We have already analyzed the effects of a cathepsin protease inhibitor, CA-074Me, on the Eco-MLV infection in NIH3T3, XC, and TE671/mCAT1 cells, and reported that the treatment efficiently and similarly suppresses the Eco-MLV vector infection, but moderately attenuated the VSV-MLV vector infection [14]. These results indicate that the Eco-MLV infection in XC cells does not require endosome acidification, but does cathepsin proteases.

### Conditioned media induce pH-independent infection in NIH3T3 cells

It has been postulated that secreted cathepsins are involved with the pH-independent Eco-MLV infection in XC cells [13]. To assess the hypothesis, we analyzed the effects of conditioned media of XC, NIH3T3, NP2, and TE671 cells on the pH-dependent MLV infection in NIH3T3 cells. Optimal condition of cathepsin B and L activities is acidic. Culture supernatants of XC and TE671 cells were both yellow, suggesting that pH values of the supernatants are low. However, during dilution of the MLV vector with the conditioned media, the diluted MLV vector solution rapidly became red. pH values of the MLV vectors diluted with the conditioned media were 6.5 to 7.0, when the vectors were inoculated to target cells.

**Figure 2:**
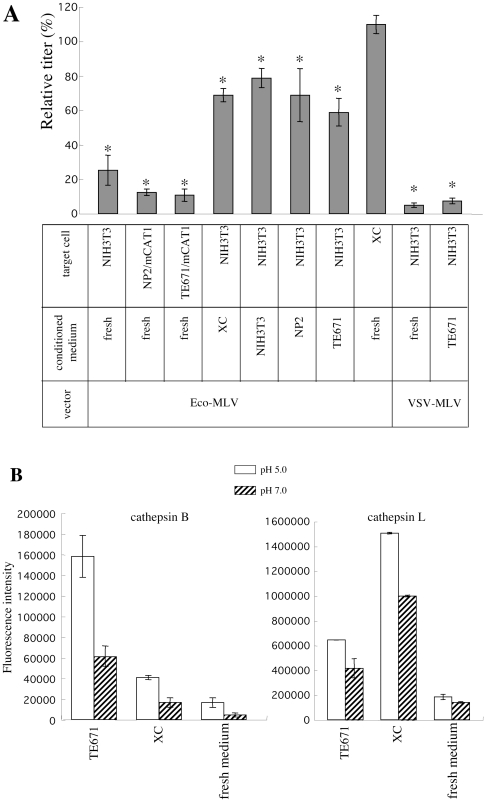
Conditioned media induce pH-independent Eco-MLV infection. (A) NIH3T3 cells were pretreated with ConA, and were inoculated with the Eco-MLV vector diluted with fresh or conditioned media. The transduction units obtained with DMSO-treated cells were set to 100%. These experiments were repeated in triplicate, and results are shown as the mean +/− SD. Asterisks indicate statistically significant differences compared to the value from the DMSO-treated cells. (B) Cathepsin B (left panel) and L (right panel) activities of TE671 and XC cell conditioned media were measured at pH 5.0 or 7.0. Fluorescence intensities were indicated. This experiment was repeated three times, and results are shown as means +/− SD.

Dilution of the Eco-MLV vector with the conditioned media of XC, NIH3T3, TE671, and NP2 cells attenuated the infection by about 1/10 (data not shown). When the MLV vector was diluted with the XC cell conditioned medium, the inhibitory effect of ConA on the Eco-MLV infection in NIH3T3 cells was diminished (Fig. 2A). The conditioned media of TE671 and NIH3T3 cells, in which the Eco-MLV infection is pH-dependent (Fig. 1A), also induced pH-independent infection in NIH3T3 cells. However, the TE671 cell conditioned medium did not abrogate the inhibitory effect of ConA on the VSV-MLV vector infection. XC and TE671 cells indeed secreted cathepsins B and L (Fig. 2B). When cathepsin B activity of the conditioned media was measured at pH 7.0, fluorescence intensities of the XC and TE671 cell conditioned media were reduced to about 1/3 time compared with those at pH 5.0, but were still higher than that of fresh medium. Cathepsin L activity of these conditioned media at pH 7.0 was moderately lower than that at pH 5.0, and was higher than that of fresh medium. These results indicate that cathepsins B and L are active at pH 7.0 in the conditioned media, and suggest that secreted cathepsins in the conditioned media induce the pH-independent Eco-MLV vector infection in NIH3T3 cells.

However, even when the Eco-MLV vector was diluted with fresh medium, ConA did not attenuate the Eco-MLV infection in XC cells (Figs. 1A and 2A). Furthermore, the conditioned media of NIH3T3, NP2, and TE671 cells, in which the Eco-MLV infection is pH-dependent, also induced the pH-independent Eco-MLV infection (Fig. 2A). Additionally, cathepsin B activity of the TE671 cell conditioned medium was higher than those of the XC cell conditioned medium (Fig. 2B). If secreted cathepsin proteases are determinant for the XC cell-specific, pH-independent Eco-MLV infection, the endosome acidification inhibitors would not suppress the Eco-MLV infection in TE671 cells. However, the inhibitors significantly attenuated the Eco-MLV infection in TE671 cells (Figs. 1A and 2A). These results indicate that secreted cathepsins are not involved in the XC cell-specific, pH-independent Eco-MLV infection.

### Secreted cathepsin proteases induce pH-independent Eco-MLV infection in NIH3T3 cells

**Figure 3:**
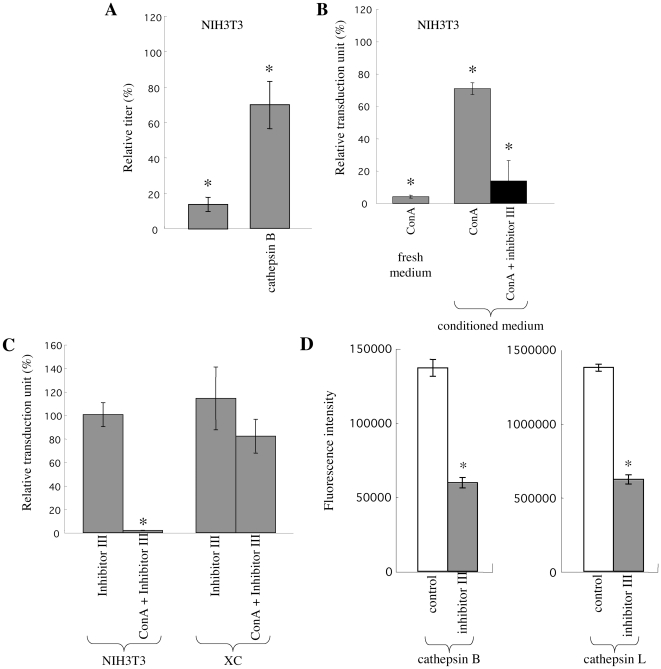
Secreted cathepsin proteases induce pH-independent Eco-MLV infection. (A) NIH3T3 cells were pretreated with ConA, and were inoculated with Eco-MLV vector diluted with fresh medium containing purified cathepsin B (100 ng/ml). (B) NIH3T3 cells were pretreated with ConA, and were inoculated with the Eco-MLV vector diluted with fresh or TE671 cell conditioned medium in the presence of cathepsin inhibitor III (200 µM). (C) NIH3T3 and XC cells were pretreated with ConA (3 nM) or cathepsin inhibitor III (200 µM). The treated cells were inoculated with Eco-MLV vector diluted with fresh medium in presence of cathepsin inhibitor III (200 µM). The transduction units obtained with DMSO-treated cells were set to 100%. These experiments were repeated in triplicate, and results are shown as the mean +/− SD. Asterisks indicate statistically significant differences compared to the value from the DMSO-treated cells. (D) Cathepsin B (left panel) and L (right panel) activities of TE671 cell conditioned medium in absence or presence of cathepsin inhibitor III were measured. Fluorescence intensities were indicated. This experiment was repeated three times, and results are shown as means +/− SD.

The above results suggested that conditioned media induce the pH-independent Eco-MLV infection by secreted cathepsin proteases. To assess this assumption, we analyzed the effects of purified cathepsin B (100 ng/ml) on the pH-dependent Eco-MLV infection in NIH3T3 cells. It has been reported that addition of cathepsin B at similar concentration enhances the Eco-MLV infection in cathepsin B-negative cells [13]. Although addition of cathepsin B reduced the Eco-MLV vector infection by about 1/5 (data not shown), it diminished the inhibitory effect of ConA on the Eco-MLV vector infection in NIH3T3 cells (P<0.05) (Fig. 3A). This result shows that addition of cathepsin B induces the pH-independent Eco-MLV infection in NIH3T3 cells.

To address whether the conditioned medium abrogates the inhibitory effect of ConA on the Eco-MLV infection by secreted cathepsins, we analyzed the effects of cathepsin inhibitor III suppressing both cathepsins B and L on the conditioned medium-induced pH-independent Eco-MLV infection in NIH3T3 cells. ConA-pretreated NIH3T3 cells were inoculated with the Eco-MLV vector diluted with the TE671 cell conditioned medium in presence of cathepsin inhibitor III. Cathepsin inhibitor III at 200 µM significantly suppressed the induction of pH-independent infection by the conditioned medium (Fig. 3B). Because cathepsin inhibitor III is not membrane permeable, the treatment of target cells at 200 µM did not attenuate infection of NIH3T3 cells by the Eco-MLV vector diluted with fresh medium (Fig. 3C). Cathepsin inhibitor III indeed attenuated activities of cathepsin proteases B and L in the TE671 cell conditioned medium (Fig. 3D). We have previously reported that a membrane unpermeable cathepsin L inhibitor, CLIK148, attenuates the Eco-MLV infection at 1000 µM [14], suggesting that much higher concentration of the unpermeable cathepsin inhibitor III is required for inhibition of the Eco-MLV vector infection than the permeable cathepsin inhibitor CA-074Me. These results indicate that secreted cathepsin proteases present in the conditioned media induce the pH-independent Eco-MLV infection in NIH3T3 cells.

### Secreted cathepsins are not involved in the XC cell-specific, pH-independent Eco-MLV infection

Even when the Eco-MLV vector was diluted with fresh medium, the Eco-MLV vector infection was pH-independent in XC cells. To assess whether cathepsins secreted from XC cells are involved in the XC cell-specific, pH-independent Eco-MLV infection, we analyzed the effect of cathepsin inhibitor III on the infection of XC cells by the Eco-MLV vector diluted with fresh medium. XC cells were inoculated with the diluted Eco-MLV vector, in the presence of cathepsin inhibitor III (200 µM). However, cathepsin inhibitor III did not affect the infection of XC cells by the Eco-MLV vector diluted in fresh medium (Fig. 3C). When we proposed that secreted cathepsins induce the pH-independent Eco-MLV infection in XC cells, we thought that the Eco-MLV infection would occur at the plasma membrane in XC cells. However, since cathepsin inhibitor III attenuated the cathepsin activity in the TE671 cell conditioned medium (Fig. 3D), but did not affect the Eco-MLV infection in XC cells, the Eco-MLV infection in the treated XC cells probably occurs through a different pathway than cell surface entry. It is most likely that the Eco-MLV vector infection in the cathepsin inhibitor III-treated XC cells occurs through acidic endosomes, since cathepsins are required for the Eco-MLV infection in XC cells [14], and because active cathepsin proteases are primarily localized to acidic endosomes. Since CA-074Me significantly inhibited the Eco-MLV vector infection in XC cells [14], the Eco-MLV infection does not occur through a cathepsin-independent pathway(s). Therefore, cathepsin inhibitor III should confer the Eco-MLV infection pathway from the cell surface to the acidic endosomes, and thus no change in the Eco-MLV vector titers should occur, if the assumption is correct. To assess whether the Eco-MLV infection of XC cells in the presence of cathepsin inhibitor III occurs through acidic endosomes, ConA-pretreated XC cells were inoculated with the Eco-MLV vector in the presence of cathepsin inhibitor III. However, the treatment of XC cells did not attenuate the Eco-MLV infection (Fig. 3C), showing that the Eco-MLV infection of XC cells in the presence of cathepsin inhibitor III does not require endosome acidification. Taken together, these results indicate that the assumption is not correct, and suggest that secreted cathepsins are not involved in the XC cell-specific, pH-independent Eco-MLV infection.

### Ampho-, Poly-, Xeno-, and XMRV-MLV vector infections occur through acidic endosomes and require cathepsin proteases in TE671 cells

**Figure 4:**
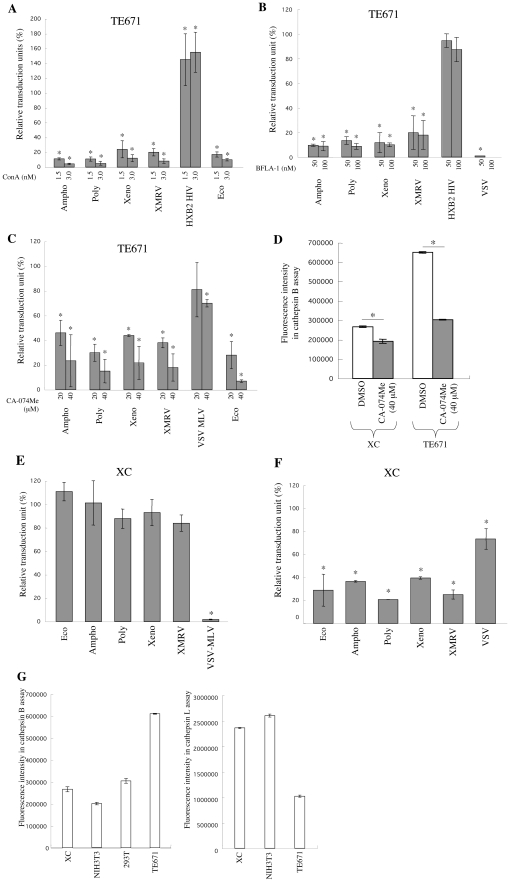
Ampho-, Poly-, Xeno-, and XMRV-MLV vector infections require endosome acidification and cathepsin proteases. (A) TE671, TE671/mCAT1, or TE671/CD4 cells were pretreated with ConA for 5 h and were inoculated with the Eco-, Ampho-, Poly-, Xeno-, XMRV-MLV, or HXB2-HIV-1 vector. (B) TE671 or TE671/CD4 cells were pretreated with BFLA-1, and were inoculated with the Ampho-, Poly-, Xeno-, VSV-, XMRV-, VSV-MLV, or HXB2-HIV-1 vector. (C) TE671 or TE671/mCAT1 cells were pretreated with CA-074Me and were inoculated with the Eco-, Ampho-, Poly-, Xeno-, VSV-, or XMRV-MLV vector. The transduction units obtained with DMSO-treated cells were set to 100%. These experiments were repeated in triplicate, and results are shown as the mean +/− SD. Asterisks indicate statistically significant differences compared to the value from the DMSO-treated cells. (D) Cathepsin B activity of cells lysates from CA-074Me-treated cells was measured. Fluorescence intensities are shown. XC cells were treated with ConA (panel E) or CA-074Me (panel F), and were inoculated with the Eco-, Ampho-, Poly-, Xeno-, XMRV-, or VSV-MLV vector. These experiments were repeated in triplicate, and results are shown as the mean +/− SD. Asterisks indicate statistically significant differences compared to the value from the DMSO-treated cells. (G) Activities of cathepsins B (left panel) and L (right panel) were measured in cell lysates from XC, NIH3T3, 293T, and TE671 cells. Fluorescence intensities were indicated. These experiments were repeated three times, and results are shown as the mean +/− SD.

It has already been reported that endosome acidification inhibitors attenuate the Eco- and Ampho-MLV infections, suggesting that these infections occur through acidic endosomes [11], [12]. To assess whether the Poly-, Xeno-, and XMRV-MLV vector infections occur through acidic endosomes, the effects of endosome acidification inhibitors, ConA and BFLA-1, on these MLV vector infections were analyzed. Human TE671 cells were pretreated with these inhibitors for 5 h, and were inoculated with the MLV vectors in absence of the inhibitors. All of these MLV vector infections were efficiently attenuated by the ConA (Fig. 4A) or BFLA-1 treatment (Fig. 4B). The BFLA-1 treatment of TE671 cells expressing human CD4 did not affect infection by an HIV-1 vector carrying the Env protein of the CXCR4-tropic HXB2 HIV-1 strain. The ConA treatment enhanced the transduction efficiency of this virus as previously reported [20], [21], suggesting that the HIV-1 vector infection do not require endosome acidification, and attenuation of the MLV infections by the inhibitors is not due to their suppressing effect on cell viability. These findings indicate that the Ampho-, Poly-, Xeno-, and XMRV-MLV vector infections all require endosome acidification in human TE671 cells.

The Eco-MLV infection requires endosomal cathepsin protease B or L [13], [14]. To assess whether the Ampho-, Poly-, Xeno-, and XMRV-MLV vector infections also require cathepsin proteases, the effect of CA-074Me on these MLV vector infections were analyzed. CA-074Me is membrane permeable and inhibits both cathepsins B and L [14], [22]. TE671 cells were pretreated with CA-074Me for 5 h, and were inoculated with the MLV vectors diluted with fresh medium. The MLV vector infections were efficiently attenuated by CA-074Me in a dose-dependent manner, but the VSV-G-MLV vector infection was not significantly inhibited (Fig. 4C). Biochemical study also revealed that the CA-074Me treatment indeed attenuated cellular cathepsin B activity in TE671 cells (Fig. 4D). These results indicate that the Eco-, Ampho-, Poly-, Xeno-, and XMRV-MLV vector infections of TE671 cells occur through acidic endosomes and require cathepsin proteases.

### pH-independent MLV infection in XC cells requires endosomal cathepsin proteases

The Eco-MLV infection of XC cells does not require endosome acidification. To assess whether the Ampho-, Poly-, Xeno-, and XMRV-MLV vector infections of XC cells also lack this requirement, the effect of ConA on these MLV vector infections was analyzed. While ConA treatment did not inhibit these MLV infections in XC cells (Fig. 4E), it did inhibit the VSV-MLV infection, indicating that the ConA treatment suppressed endosome acidification. These results indicate that the Eco-, Ampho-, Poly-, Xeno-, and XMRV-MLV vector infections of XC cells are all independent of endosome acidification.

If the MLV infections of XC cells do not occur through acidic endosomes, it may not require cathepsin proteases, because secreted cathepsins are not involved in the pH-independent infection of XC cells and these proteases primarily exist in acidic endosomes. However, CA-074Me treatment inhibited the Eco-MLV infection in XC cells as well as in NIH3T3 cells [14]. The Ampho-, Poly, Xeno-, and XMRV-MLV infections in XC cells were also suppressed by CA-074Me (Fig. 4F). CA-074Me treatment indeed decreased cathepsin B activity in XC cells to that in untreated NIH3T3 cells in which cathepsin B activity is negative or extremely low [14] (Figs. 4D and G). These results suggest that the MLV infections in XC cells are pH-independent but require endosomal cathepsin proteases.

### Cathepsin proteases are activated in the absence of endosome acidification in XC cells

**Figure 5:**
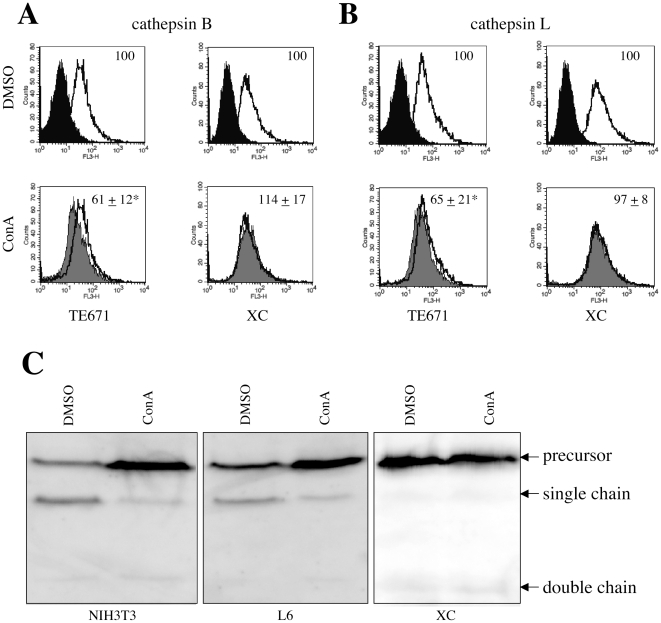
Treatment with endosome acidification inhibitors does not alter activities of cathepsins B and L in XC cells. TE671 and XC cells were treated with ConA (3 nM) or DMSO. The treated cells were stained with the cathepsin B (panel A) or L (panel B) detection reagent. Closed areas indicate cells unstained with the reagents. Open areas indicate cells treated with DMSO and stained with the reagent. Grey areas indicate cells treated with ConA and stained with the reagent. Means of fluorescent intensity obtained with DMSO-treated cells were set to 100%. These experiments were repeated in triplicate, and results are shown as the mean +/− SD. Asterisks indicate statistically significant differences compared to the DMSO-treated cells. Mouse NIH3T3, rat L6, and XC cells were treated with DMSO or ConA (3 nM). Cell lysates from the treated cells were analyzed by Western immunoblotting using an anti-mouse cathepsin L ancbody (panel C).

Because cathepsin proteases are activated by low pH in acidic endosomes [23], endosome acidification inhibitors may inhibit MLV infections by suppressing cathepsin protease activation. To address this hypothesis, the effect of ConA treatment on activities of cathepsins B and L in living cells were measured. ConA treatment attenuated cathepsin B (Fig. 5A) and L (Fig. 5B) activities in TE671 cells (P<0.05), but did not in XC cells, suggesting that cathepsin proteases are activated without endosome acidification in XC cells.

Cathepsin B and L proteases are maturated by their processing in acidic condition. To assess whether the acidification inhibitor affects the cathepsin protease processing, Western immunoblotting of cell lysates prepared from ConA-or DMSO-treated cells was performed using an anti-mouse cathepsin L antibody. In mouse NIH3T3 and rat L6 cells in which the Eco-MLV infection is pH-dependent (Fig. 1B), levels of the cathepsin L precursor and processed single chain proteins were elevated and reduced by the ConA treatment, respectively, indicating that the ConA treatment suppresses the cathepsin L processing. In contrast, levels of the cathepsin L precursor and processed proteins (single and double chains) were not affected by the ConA treatment in XC cells, suggesting that ConA treatment does not attenuate cathepsin L activity in XC cells. Interestingly, the cathepsin L processing occurs less efficiently in XC cells than in NIH3T3 and L6 cells, although the cathepsin L activity in XC cells was similar to that in NIH3T3 cells (Fig. 4G). Cathepsin L cDNA was isolated from XC cells by RT-PCR (Accession No. AB630323). The XC cell cathepsin L has no change in amino acid sequence compared to already reported rat cathepsin L sequences. Cathepsin B protein was not detected in XC cells by Western immunoblotting, probably because cathepsin B activity was relatively lower in XC cells (Fig. 4G). Finally, these results indicate that cathepsins B and L are activated without endosome acidification in XC cells.

### MLV infections require dynamin-mediated endocytosis in NIH3T3, XC, and TE671 cells

**Figure 6:**
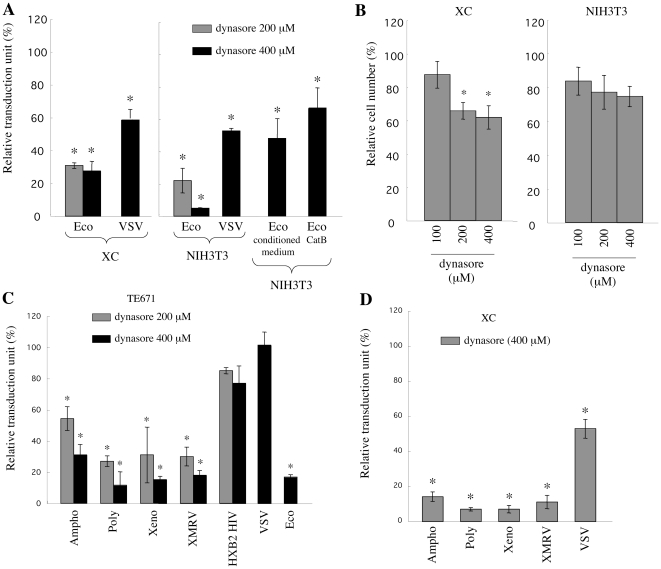
MLV infections are inhibited by dynasore. (A) XC and NIH3T3 cells were pretreated with dynasore and were inoculated with the Eco-MLV vector diluted with fresh, TE671 cell conditioned, or cathepsin B (100 ng/ml)-containing medium. (B) XC and NIH3T3 cells were treated with dynasore, washed, and cultured for 48 h. Cells resistant to trypan blue staining were quantified. (C) TE671, TE671/mCAT1, or TE671/CD4 cells were pretreated with dynasore and were inoculated with the Eco-, Ampho-, Poly-, Xeno-, XMRV-, VSV-MLV, or HXB2-HIV-1 vector. (D) XC cells were pretreated with dynasore and were inoculated with the Ampho-, Poly-, Xeno-, XMRV-, or VSV-MLV vector. The transduction units obtained with DMSO-treated cells were set to 100%. These experiments were repeated in triplicate, and results are shown as the mean +/− SD. Asterisks indicate statistically significant differences compared to the value from the DMSO-treated cells.

The above findings prompted us to speculate that these MLV infections in XC cells occur through endosomes but do not require endosome acidification, because cathepsin proteases are activated independently of endosome acidification. To address this hypothesis, the effect of an endocytosis inhibitor, dynasore, on the MLV infections was analyzed. Dynasore is an inhibitor of dynamin GTPase required for endocytosis [24]. Target cells were pretreated with dynasore for 5 h and were inoculated with the Eco-MLV vector diluted with fresh medium. The dynasore treatment similarly attenuated the Eco-MLV infection in XC and NIH3T3 cells (Fig. 6A). The inhibitory effect of dynasore on cell viability (Fig. 6B) was much more modest than the inhibition of the Eco-MLV infection. The dynasore treatment also inhibited the Ampho-, Poly-, Xeno-, and XMRV-MLV infections in TE671 (Fig. 6C) and XC cells (Fig. 6D). However, dynasore treatment of CD4-expressing TE671 cells did not inhibit infection by the HIV-1 vector containing the HXB2 Env protein, consistent with previous reports [20], [21], [25], [26]. The VSV-G-mediated infection was moderately inhibited by dynasore in XC and NIH3T3 cells, but was not affected in TE671 cells (Fig. 6C). These results suggest that the Eco-, Ampho-, Poly-, Xeno-, and XMRV-MLV infections in XC cells require the dynamin-dependent endocytosis.

When NIH3T3 cells were inoculated with the Eco-MLV vector diluted with the TE671 cell conditioned or cathepsin B-containing medium, the inhibitory effect of ConA on the Eco-MLV infection was minimized (Figs. 2A and 3A), suggesting that the Eco-MLV infection in the presence of secreted cathepsin proteases occurs at the plasma membrane. To assess this hypothesis, the effect of dynasore on infection by the Eco-MLV vector diluted with the TE671 cell conditioned or cathepsin B-containing medium was analyzed. As expected, the infection was not significantly suppressed by dynasore (P<0.05) (Fig. 6A). This result supports that the Eco-MLV vector infection in the presence of secreted cathepsin proteases occurs at the plasma membrane.

**Figure 7:**
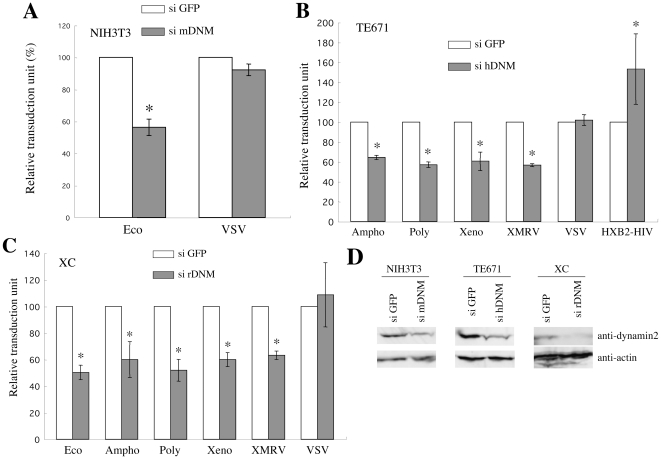
MLV infections are inhibited by siRNA-mediated knockdown of dynamin 2 expression. (A) NIH3T3 cells were transfected with an siRNA against mouse dynamin 2 mRNA, and were inoculated with the Eco-MLV vector. (B) TE671 or TE671/CD4 cells were transfected with an siRNA against human dynamin 2 mRNA, and were inoculated with the Ampho-, Poly-, Xeno-, XMRV-, VSV-MLV, or HXB2-HIV-1 vector. (C) XC cells were transfected with an siRNA against rat dynamin 2 mRNA, and were inoculated with the Eco-, Ampho-, Poly-, Xeno-, XMRV- or VSV-MLV vector. The transduction units obtained with DMSO-treated cells were set to 100%. These experiments were repeated in triplicate, and results are shown as the mean +/− SD. Asterisks indicate statistically significant differences compared to the value from the DMSO-treated cells. (D) Dynamin 2 protein expression in the siRNA-transfected cells was analyzed by Western immunoblotting using an anti-dynamin 2 antibody. Actin expression was analyzed as control.

To confirm that the MLV infections occur through endosomes even in XC cells, we analyzed the effects of siRNAs against dynamin 2 mRNA on these MLV infections. Because mouse NIH3T3, human TE671, and rat XC cells were used in this study, we constructed siRNAs against mouse, human, and rat dynamin 2 mRNAs. As expected, the knockdown of dynamin 2 expression mediated by the siRNAs suppressed the MLV vector infections in mouse NIH3T3 (Fig. 7A), human TE671 (Fig. 7B), and rat XC cells (Fig. 7C), but not the CXCR4-tropic HIV-1 and VSV infections. The siRNAs indeed attenuated dynamin 2 expression analyzed by Western immunoblotting (Fig. 7D). These results indicate that the Eco-, Ampho-, Poly-, Xeno-, and XMRV-MLV vector infections require dynamin 2-mediated endocytosis even in XC cells.

### Ebola virus infection is pH-independent in XC cells

**Figure 8:**
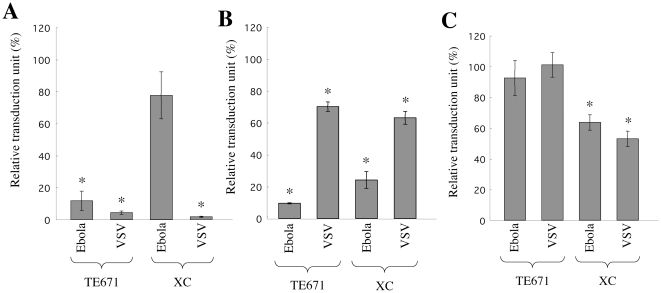
Ebola virus infection in XC cells is pH-independent. TE671 and XC cells were pretreated with ConA (panel A), CA-074Me (panel B), or dynasore (panel C), and were inoculated with the Ebola virus GP- or VSV-G-pseudotyped MLV vector. The transduction units obtained with DMSO-treated cells were set to 100%. These experiments were repeated in triplicate, and results are shown as the mean +/− SD. Asterisks indicate statistically significant differences compared to the value from the DMSO-treated cells.

Ebola virus infection also requires endosome acidification and endosomal cathepsin proteases like the MLV infections [27]. Infection of XC cells by Ebola virus GP-pseudotyped MLV vector might be low pH-independent. To assess this hypothesis, the effect of ConA treatment on the Ebola-MLV vector infection was analyzed. As expected, the ConA treatment inhibited the Ebola virus GP-mediated infection in TE671 cells, but not in XC cells (Fig. 8A). The Ebola virus GP-mediated infection was suppressed by CA-074Me treatment in TE671 and XC cells (Fig. 8B), indicating that the Ebola-MLV vector infection in XC cells requires endosomal cathepsins but not endosome acidification. However, dynasore treatment did not significantly attenuated the Ebola-MLV infection in TE671 and XC cells (Fig. 8C), consistent with the previous report showing that the Ebola virus infection occurs through dynamin-independent macropinocytosis [28]. These results indicate that not only the MLV infections but also the Ebola virus infection are pH-independent in XC cells.

### ConA, CA-074Me, dynasore, siRNAs do not inhibit MLV vector binding to target cells

**Figure 9:**
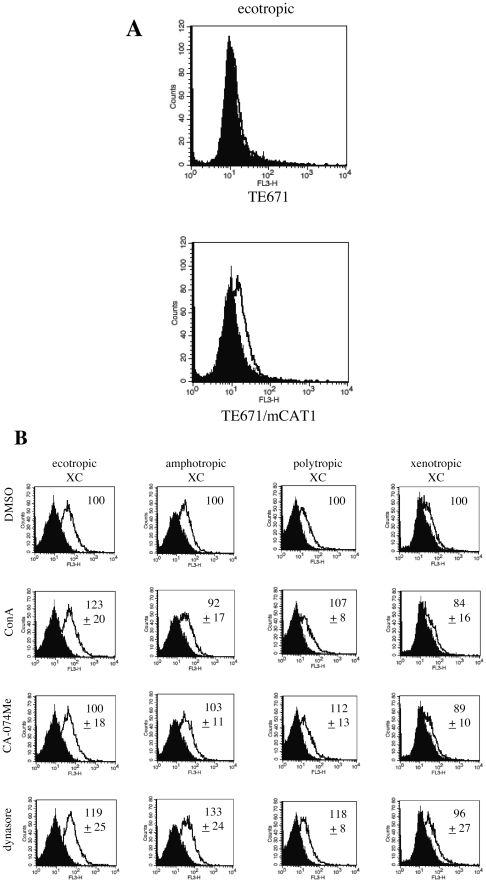
Binding of the MLV vectors to target cells is not affected by ConA, CA-074Me, or dynasore treatments. (A) Binding of the Eco-MLV vector particles to TE671 (upper panel) and TE671/mCAT1 (lower panel) was analyzed. (B) XC cells were pretreated with DMSO, ConA, CA-074Me, or dynasore, and were incubated with the Eco-, Ampho-, Poly-, or Xeno-MLV vector. The fluorescence intensities obtained with DMSO-treated cells were set to 100%. These experiments were repeated in triplicate, and values are shown as the mean +/− SD.

To test whether MLV vector binding was modulated by ConA, CA-074Me, or dynasore inhibitor, we monitored the vector-cell complex formation in the presence of each inhibitor. The antiserum used for this assay recognizes Env proteins of Eco-, Ampho-, Poly, and Xeno-MLVs in Western immunoblotting [29]. The Eco-MLV vector bound to TE671/mCAT1 cells but not to TE671 cells (Fig. 9A), indicating that the binding is mCAT1-dependent, as previously reported in human NP2 cells [30]. Binding of the Eco-MLV vector to target cells was not suppressed by the inhibitors in XC (Fig. 9B), NIH3T3, and TE671 (data not shown) cells. Similarly, the siRNA-mediated knockdown of dynamin 2 expression did not inhibit the formation of MLV/target cell complexes (data not shown). Thus, the inhibitors and the siRNA-mediated dynamin 2 knockdown did not attenuate the MLV vector infections by suppressing MLV particle binding to the target cells.

### Digestion of MLV Env proteins by cathepsin B

**Figure 10:**
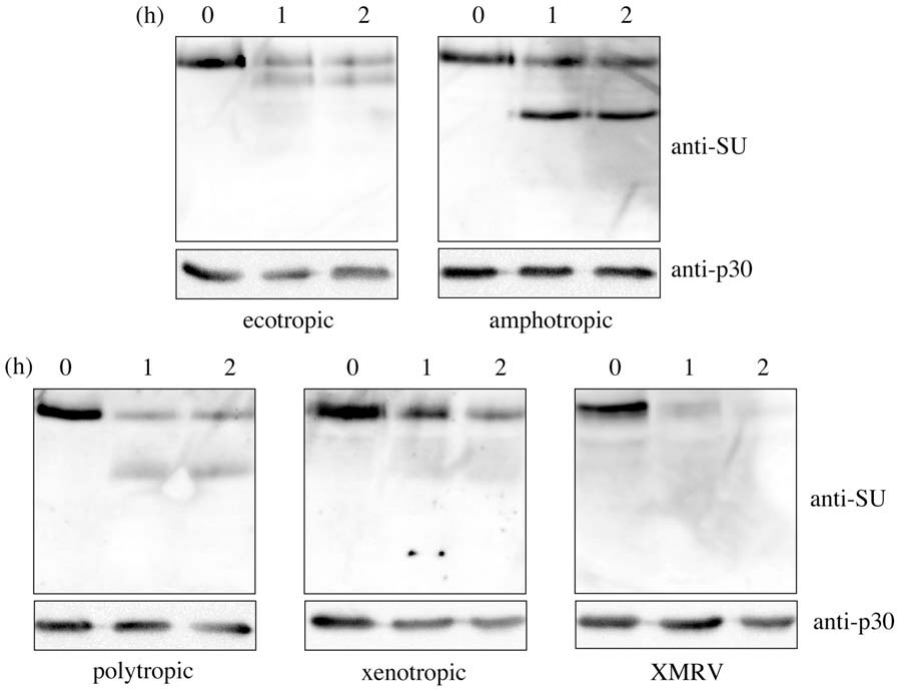
Digestion of MLV Env proteins by cathepsin B. The MLV vector particles were treated with cathepsin B (100 ng/ml). The products were analyzed by Western immunoblotting using the anti-MLV SU antiserum.

This study indicates that cathepsin proteases are required for the Eco-, Ampho-, Poly-, Xeno-, and XMRV-MLV vector infections. It has been reported that the Eco-MLV Env protein is digested by cathepsin B [13], suggesting that the digestion of the Env protein activates the entry capability. To assess whether the Ampho-, Poly-, Xeno-, and XMRV Env proteins are digested by cathepsin B, purified MLV vector particles were treated with cathepsin B (100 ng/ml) at pH 5.0, and analyzed by Western immunoblotting. Digested products were detected in the Eco-, Ampho-, and Poly-Env proteins (Fig. 10). It has been reported that 52 and 28 kDa digested products were detected in digestion of the Eco-Env protein by cathepsin B, and the 28 kDa peptide contains N-terminal receptor-binding domain [13]. In our study, the 52 kDa product was detected, but the 28 kDa was not. Because the anti-MLV SU antiserum used in our study recognized all of the Eco-, Ampho-, Poly-, Xeno-, and XMRV-Env proteins, the antiserum primarily recognizes the conserved C-terminal domains of these Env proteins, but not the variable N-terminal receptor binding domains. Therefore, the 28 kDa product was not detected by this antiserum. Digested products were not detected in the Xeno- and XMRV-Env proteins. However, amounts of the undigested SU proteins were reduced by the cathepsin B treatment. This result suggests that the Xeno- and XMRV-Env proteins are digested by cathepsin B at many cleavage sites.

## Discussion

The XC cell-specific, pH-independent MLV infection is one of the well-recognized mysteries in the MLV study field, but the mechanism had not been elucidated for long time. It had been generally accepted that the Eco-MLV entry into XC cells occurs at the plasma membrane, and not through endosomes [11]. However, the CA-074Me treatment attenuated MLV infections in XC cells, showing that the MLV infections of XC cells require endosomal cathepsin proteases. This conflicting result prompted us to challenge the previous theory that the Eco-MLV infection in XC cells occurs at the plasma membrane [11].

**Figure 11:**
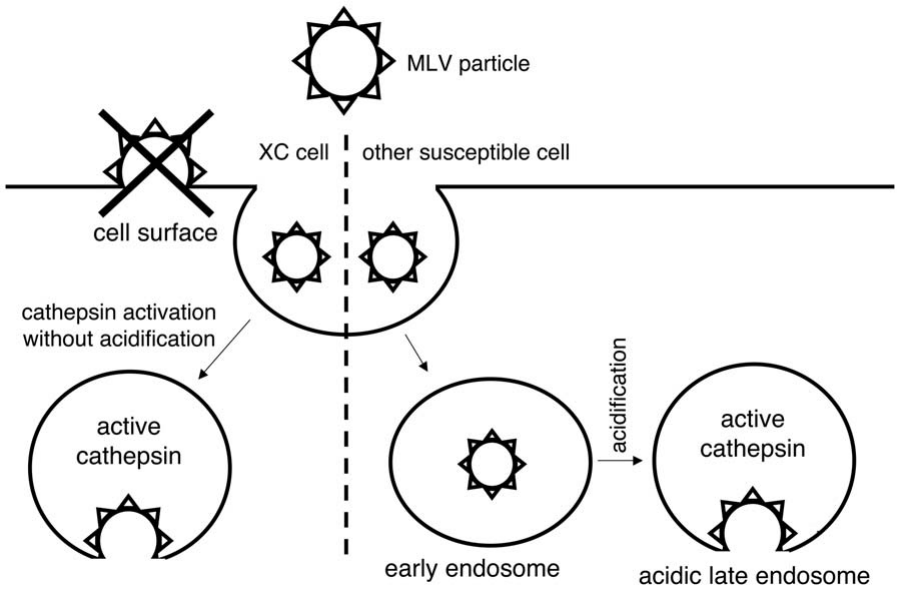
Entry pathways of MLV infection in XC cells and in other susceptible cells.

The propeptide of cathepsin proteases facilitates folding of the enzymes, acts as an inhibitor preventing proteolytic activity of the enzymes, and is responsible for targeting to endosomes [23]. The propeptide of cathepsin protease masks its active site, and inhibits the protease activity. Conformation of the propeptide-containing cathepsin protease is changed in acidic condition of endosomes, and the active site is exposed. After the active site is exposed by low pH, the propeptide of cathepsin is cleaved by itself or other proteases to form a fully activated cathepsin protease. Therefore, it is thought that treatment by the endosome acidification inhibitors prevent the MLV infections by suppressing the cathepsin protease activation. The treatment did not inhibit cathepsin protease activity and cathepsin L processing in XC cells, indicating that cathepsin proteases are activated independently of endosome acidification in XC cells. This independence may explain why the MLV infections in XC cells do not require endosome acidification and are not attenuated by the endosome acidification inhibitors. Thus, the MLV infections in XC cells occur through endosomes and the pH-independent activation of endosomal cathepsin proteases induces the pH-independent MLV infections in XC cells (Fig. 11). The dynasore treatment and siRNA-mediated dynamin 2 knockdown inhibited the MLV infections in all examined cells, strongly supporting the above observations. These results indicate that the MLV particles are internalized by endocytosis for a productive infection in XC cells similar to other susceptible cells.

The MLV and Ebola virus Env proteins used in this study recognize different infection receptors as described in Introduction. It is suggested that interactions with these different receptors lead to the same endocytic entry pathway for all these viruses. Based on this model of the virus entry pathway, the virus Env proteins are activated by cathepsin-mediated digestion after internalized into acidic endosomes. Therefore, it is unlikely that viral proteins directly induce the internalization of viral particles into endosomes. This suggests that these different infection receptors are responsible for the endocytic entry pathway.

The conditioned media of XC, TE671, and NIH3T3 cells contained cathepsin protease activity, and induced the pH-independent MLV vector infection in NIH3T3 cells. Because the MLV vector-producing TELCeB6 cells were constructed from TE617 cells [31], their cells secrete cathepsin proteases as well as the MLV vector particles. Env proteins on the MLV vector particles should be digested by secreted cathepsin proteases, soon after the vector particles are released from the vector-producing cells. However, infection by the MLV vectors produced from TELCeB6 cells required endosome acidification and cathepsin proteases in NIH3T3 cells. Dilution of the MLV vector with the conditioned or cathepsin B-containing medium significantly attenuated the transduction efficiency, suggesting that the digested Env proteins are unstable or released from the MLV vector particles. Indeed, the digested products of Env proteins were not detected in the MLV vector preparations. Therefore, infections by the MLV vectors produced from transfected TELCeB6 cells require endosome acidification and cathepsin proteases in NIH3T3 cells.

Cathepsin L activity in XC cells was similar to that in NIH3T3 cells, and cathepsin L processing occurred less efficiently in XC cells than in NIH3T3 and L6 cells, suggesting that unprocessed cathepsin L is active in XC cells. It has been reported that glycosaminoglycans such as chondroitin sulfate and heparin facilitate cathepsin B activation at natural pH [23]. However, treatment of XC cells with chondroitinase or heparinase did not affect the pH-independent Eco-MLV vector infection (data not shown). Mechanism by which cathepsin proteases are activated without endosome acidification in XC cells is unknown. It has been reported that disruption of microtubules markedly reduces the Eco-MLV infection in NIH3T3 cells, but does not in XC cells [19]. Based on the previous theory that the Eco-MLV infection occurs at cell surface in XC cells but does through endosomes in NIH3T3 cells, the authors have explained that the difference is resulted from the different entry pathways. However, since it was found that the Eco-MLV infection occurs through endosomes in XC and NIH3T3 cells, endosomes in which cathepsin proteases are activated without acidification might be different from those in which cathepsins are activated by acidification. Microtubule-independent endocytosis might induce endosomes in which cathepsins are activated without acidification, and specifically occur in XC cells.

The endosome acidification inhibitors significantly (1/10) attenuated the MLV, vector infections, but moderately (1/2) suppressed cathepsin activities. MLV vectors were diluted to induce about 20 to 40 infected cells in 1 mm^2^. Because 6-cm dishes were used in this experiment, it was estimated that about 10^5^ cells are infected in a dish. In the inhibitor-treated cells, 10^4^ cells were still infected. Therefore, it is not suspicious that cathepsin activity of the target cells correlates to susceptibility to the MLV infections.

The membrane-permeable CA-074Me treatment inhibited the MLV infections at 40 µM, but the unpermeable cathepsin inhibitor III did not even at 200 µM. We have previously reported that an unpermeable cathepsin L inhibitor, CLIK148, suppresses the Eco-MLV infection at 1000 µM [14]. All these inhibitors can be incorporated into endosomes by endocytosis. Additionally, the membrane-permeable inhibitor can reach endosomes by passing through membranes. Therefore, the membrane-permeable inhibitor suppressed the MLV vector infections at lower concentration than the unpermeable inhibitors.

In this study, the VSV-G-pseudotyped MLV vector infection was moderately suppressed by dynasore in XC and NIH3T3 cells, and was not in TE671 cells. The knockdown of dynamin 2 expression by siRNAs did not affect the VSV-G-pseudotyped vector infection. Although the VSV-G-mediated infection also occurs through endosomes, it has been reported that VSV infection enters host cells through endosomes incompletely coated with clathrin [32]. The unconventional endocytosis might not require dynamin 2, and the VSV-G-mediated infection might be independent of dynamin 2.

Because the Eco-MLV infection is pH-independent and induced pH-independent syncytium formation in XC cells, it had been widely accepted that the Eco-MLV infection in XC cells occurs at cell surface [11]. It had been thought that the pH-independent Eco-MLV infection always links to the pH-independent syncytium formation by the Eco-MLV infection. However, Wilson et al have found a transformed cell line, NIH3T3/DTras, in which the Eco-MLV infection is pH-dependent but induces the pH-independent syncytium formation [33], suggesting that the membrane fusion mechanisms for the viral entry and for syncytium formation are different, consistent with our previous finding [8].

It had been generally accepted that the Eco-MLV entry into XC cells occurs at the plasma membrane, and not through endosomes [11]. We believe that our study resolved the mechanism by which the MLV infections are pH-independent in XC cells at last. The conclusion made in this study does not support the previous conclusion and represent the novel finding.

## Materials and Methods

### Cells

Human 293T [34], NP2 [35], and TE671 [31], mouse NIH3T3 [36] and SC-1 [37], rat XC [18] and L6 [38], and monkey COS7 [39] cells were cultured in Dulbecco's modified Eagle's medium (Wako) supplemented with 8% fetal bovine serum (Biosources) at 37°C in 5% CO_2_. MLV vector producing cells were constructed by stable transfection of TELCeB6 cells [31] with ecotropic Friend [8], amphotropic [40], polytropic [41], xenotropic [6], or XMRV [42] Env expression plasmid as previously reported [29]. Eco-MLV receptor-expressing 293T and TE671 cells were constructed using an mCAT1-encoding MLV vector as previously described [17].

### Retrovirus vectors

Culture supernatants of TELCeB6 cells [31] stably transfected with the ecotropic, amphotropic, polytropic, xenotropic, or XMRV Env protein expression plasmid were used to inoculate target cells. The VSV-G- or Ebola virus GP-pseudotyped MLV vector was constructed by transient transfection of TELCeB6 cells with a VSV-G [43] or Ebola virus GP expression plasmid [28]. The HIV-1 vector containing the HXB2 Env protein was constructed by transient transfection of COS7 cells with the packaging construct of HIV-1 [44], the LacZ-encoding HIV-1 vector genome [45], and the HXB2 Env expression plasmids as previously reported [46]. The culture media from transfected cells was replaced with fresh media 24 h after transfection, and the cells were cultured for an additional 24 h. Supernatants of transfected cell cultures were used to inoculate target cells.

### Transduction assay

Target cells were plated onto 6-cm dishes, and cultured for 24 h. The cells were pretreated with ConA (Sigma), BFLA-1 (Sigma), CA-074Me (Sigma), or dynasore (Calbiochem) for 5 h. The treated cells were washed with media to remove the inhibitors. Culture supernatants of the vector-producing cells were diluted with fresh or conditioned media as follows: the Eco- and Ampho-MLV vectors were diluted 1000-fold and the Poly-, Xeno- and VSV-MLV as well as XMRV vectors were diluted 10-fold. The Ebola virus GP-pseudotyped MLV vector was not diluted. Target cells were inoculated by complete replacement of culture medium by diluted viral vectors. By the dilution, 20–40 infected cells were detected in 1 mm^2^ of control DMSO-treated cells. Alternatively, the target cells were inoculated with the MLV vectors in presence of cathepsin inhibitor III (Calbiochem) or purified cathepsin B (Calbiochem). The inoculated cells were cultured for 48 h, and then stained with 5-bromo-4-chloro-3-indolyl-β-D-galactopyranoside (Wako). The blue cells were counted in 10 randomly selected microscopic fields, and relative values to the infected cell numbers in control cells were compared between samples.

### Cell viability

Target cells were treated with the inhibitors for 5 h and were washed with media to remove the inhibitors. Fresh media was added to the cells and they were cultured for 48 h. The cells were then collected and stained with trypan blue (Cosmo Bio). The amount of unstained cells was quantitated in a counting chamber.

### Cathepsin protease activity assay

To measure the activities of cathepsins B and L in cell lysates, the cathepsin B and L activity assay kits (BioVision, Mountain View, CA) were used, respectively. The cathepsin activity assay kit is a fluorescence-based assay that utilizes the preferred cathepsin B or L substrate amino acid sequence labeled with amino-4-trifluoromethyl coumarin (AFC). Cell lysates that contain cathepsin B or L will cleave the synthetic AFC-labeled substrate to release free AFC, and generate fluorescence. Cell lysates prepared from 1×10^6^ cells were incubated with the cathepsin B or L substrate for 1 h at 37°C, and fluorescence intensity at 505 nm was measured by a fluorescence microplate reader (PerkinElmer).

Cathepsin activities in living cells were measured as follows. Cells were stained with the cathepsin B or L detection reagent (Cell Technology, Minneapolis, MN). The reagent utilizes fluorophore cresyl violet that is bi-substituted by an amide linkage to a peptide that contains a cathepsin B or L target cleavage sequence. In this form, the cresyl violet leaving group is non-fluorescent. Following cleavage at the amide linkage site by cathepsin B or L in living cells, the mono and non-substituted cresyl violet fluorophores generate red fluorescence. The stained cells were subjected to a flow cytometer (BD Biosciences, San Jose, CA) to measure fluorescence intensity of the cells.

### siRNA-mediated knockdown of dynamin expression

Nucleotide sequences of sense strands of siRNAs against human, rat, and mouse dymanin 2 mRNAs are CCC UAC GUA GCA AAC UAC AGA, GAC UGC UGA GUC GUU GUC UUG, and GCA CCC ACA GUG UAG GAC AGU, respectively. A siRNA against green fluorescence protein (GFP) was used as a control. The nucleotide sequence of sense strand of the GFP siRNA was CUG GAG UUG UCC CAA UUC UUG. These siRNAs were designed and synthesized by RNAi Co. LTD. Cells were transfected with siRNAs (400 pmol) by TransIT TKO (12 µl) (Mirus).

### Western immunoblotting

Cell lysates were subjected to sodium dodecyl sulfate polyacrylamide gel electrophoresis (BioRad), and were transferred onto a PVDF membrane (Millipore). The membrane was treated with an anti-dynamin 2, -cathepsin L (Santa Cruz), -MLV SU, or -p30 antiserum [29], and then with a horseradish peroxidase-conjugated protein G (BioRad). The anti-MLV SU antiserum was obtained from ViroMed Biosafety Laboratories. Proteins G-bound polypeptides were visualized by ECL Western blotting detection reagents (Amersham Pharmacia Biotech).

### MLV vector binding assay

The MLV vector binding assay was performed as reported previously [30], [47]. Target cells (1×10^6^) were incubated with the MLV vector solution for 1 h at 4°C, and unbound vector particles were removed by two washes with PBS. Vector-cell complexes were incubated sequentially at 4°C with the goat anti-MLV SU antiserum and then with PE-conjugated anti-goat IgG antibody (Jackson Laboratories). Fluorescence intensity of the cells was analyzed by a flow cytometer (BD Bioscences).

### Digestion of MLV Env proteins by cathepsin B

Culture supernatants of the MLV vector producing cells were filtered through 0.45 µm membranes (MILLIPORE), and centrifuged through 20% sucrose to collect MLV particles. The MLV particles were incubated with bovine cathepsin B (Calbiochem) at 37°C, and subjested to SDS-PAGE. MLV proteins were analyzed by Western immunoblotting using the anti-MLV SU or -p30 antiserum.

### Statistical analysis

Differences between two groups of data were determined by the Student's t-test. The statistical significance was set at p<0.05 for all tests.
